# Supplementary material for: Genomic and patient epidemiology of Streptococcus dysgalactiae subspecies equisimilis in Houston, Texas
Source: Microbiol Spectr. 2026 Mar 6;14(4):e03683-25. doi: 10.1128/spectrum.03683-25 (PMC13055375; doi:10.1128/spectrum.03683-25)
Supplement: Supplemental figure legends — Legends for Figures S1 to S3. [file spectrum.03683-25-s0001.docx]

**FIG S1** Illustrated is the relationship between pair-wise core and accessory genome distances for all 865 isolates of SDSE. The calculated decision boundary was used to partition the data into 44 distinct genetic lineages (GLs). The high network transitivity indicates good within clusters/GLs cohesion, and the low network density indicates that the population is highly structured into many discrete clusters/GLs.

**FIG S2** SDSE genetic lineage recombination assessment. Illustrated in panels A-to-L are phylogenetic trees (on the left) and recombination block diagrams (on the right) with a heatmap of recombination events (along the top) as inferred with Gubbins. Below each diagram are given recombination and mutation metrics for the core chromosomal sequences analyzed as determined with ClonalFrameML. The GL and number of isolates/core chromosomal sequences analyzed is given in the header of each panel.

**FIG S3** Genetic lineage recombination metrics outlier assessment. Illustrated as dot plots are the recombination metrics determined for the SDSE cohort and genetic lineages as determined for the core chromosome sequences with ClonalFrameML. The genetic lineage core chromosome sequences analyzed are color coded as given in the index. The recombination metric is indicated below each plot. The red bar indicates the mean, and the value of the mean and standard deviation are given above each plot. Grubbs’ outlier test (i.e. Extreme Studentized Deviate test) was used to test if the metric with the greatest deviation from the mean was statistically significantly different from the others. None of the GLs had a most divergent recombination metric that was a significant outlier. Although recombination metrics varied among the GLs, the variation was not statistically significant, indicating that the GLs are evolving similarly in terms of recombination and single site mutation.
