# Supplementary figures and images for: Genomic and patient epidemiology of Streptococcus dysgalactiae subspecies equisimilis in Houston, Texas
Source: Microbiol Spectr. 2026 Mar 6;14(4):e03683-25. doi: 10.1128/spectrum.03683-25 (PMC13055375; doi:10.1128/spectrum.03683-25)

## Refined fit boundary

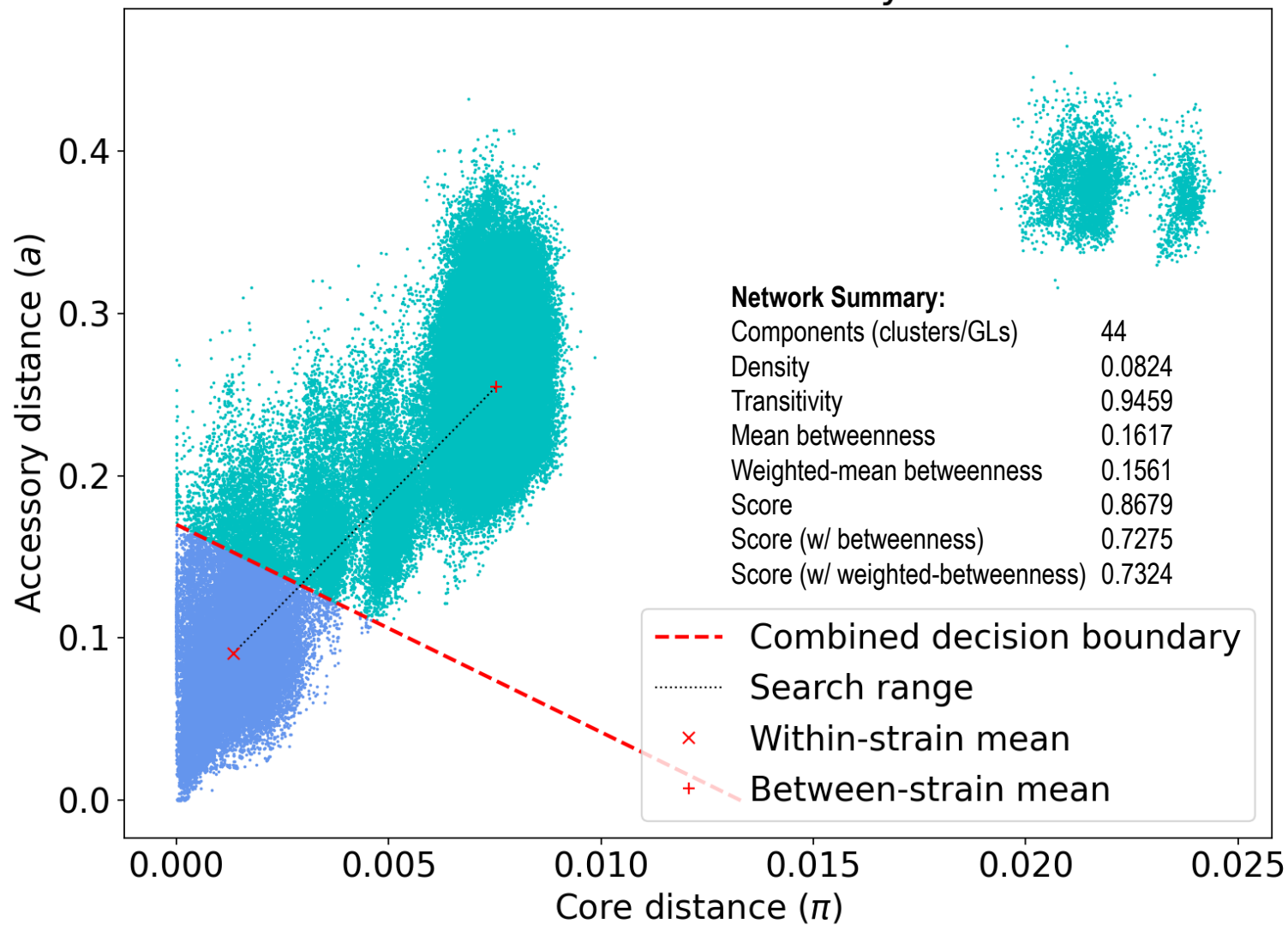

Supplement: Figure S1 — PopPUNK refined fit. [file spectrum.03683-25-s0002.pdf]

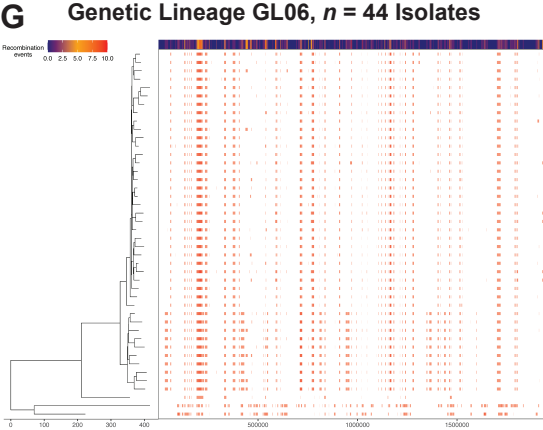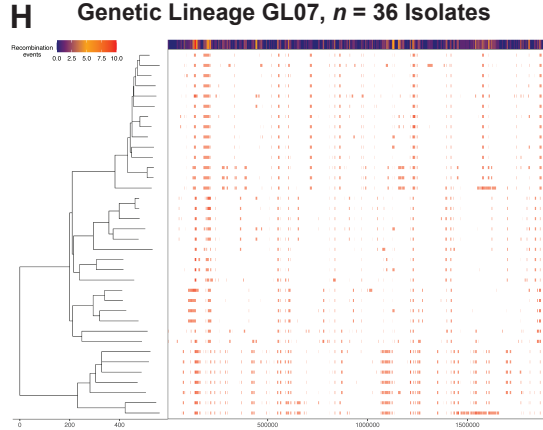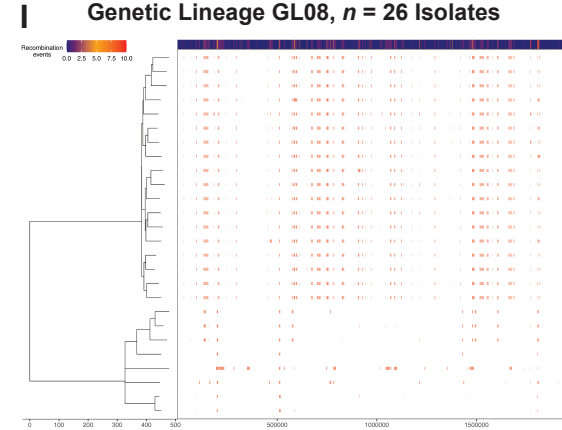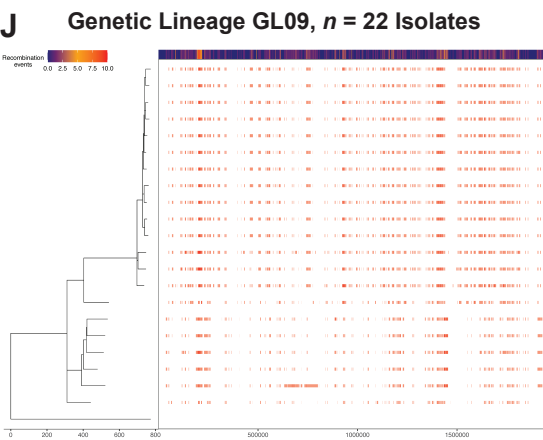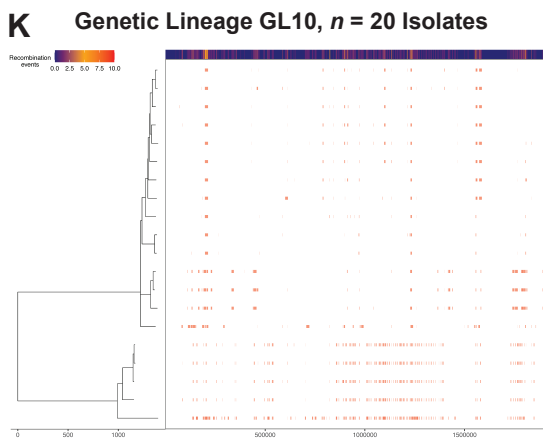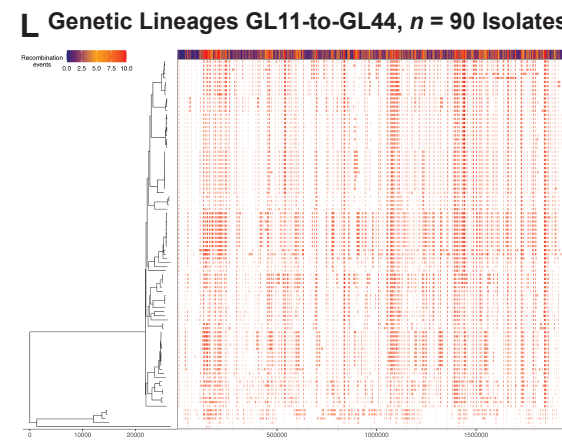

Supplement: Figure S2.2 — Recombination for GL06 to GL10. [file spectrum.03683-25-s0004.pdf]

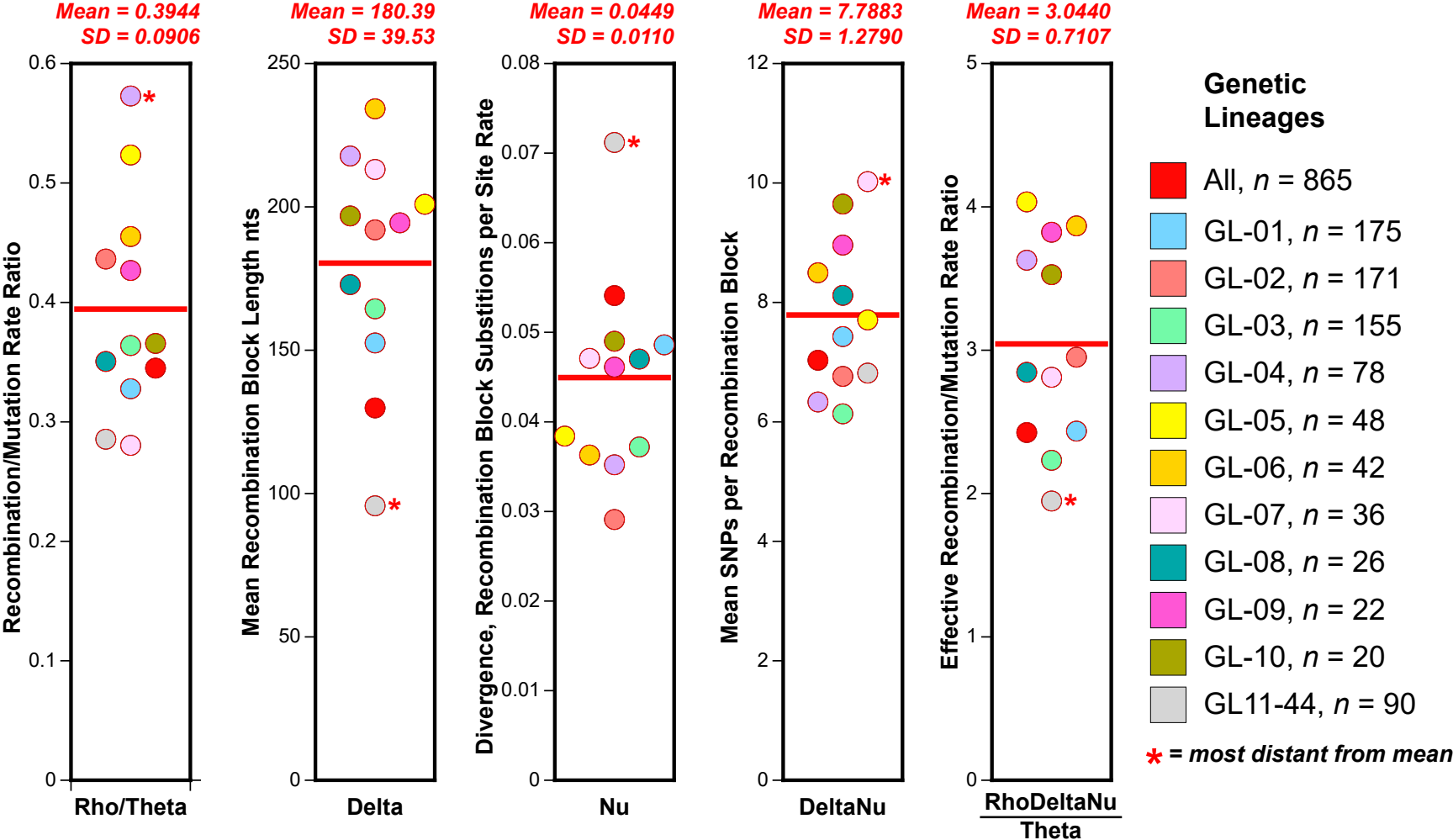

Supplement: Figure S3 — Grubb's test for recombination outlier. [file spectrum.03683-25-s0005.pdf]
