# Supplementary material for: Genomic and patient epidemiology of Streptococcus dysgalactiae subspecies equisimilis in Houston, Texas
Source: Microbiol Spectr. 2026 Mar 6;14(4):e03683-25. doi: 10.1128/spectrum.03683-25 (PMC13055375; doi:10.1128/spectrum.03683-25)
Supplement: Figure S2.1 — Recombination for GL01 to GL05. [file spectrum.03683-25-s0003.pdf]

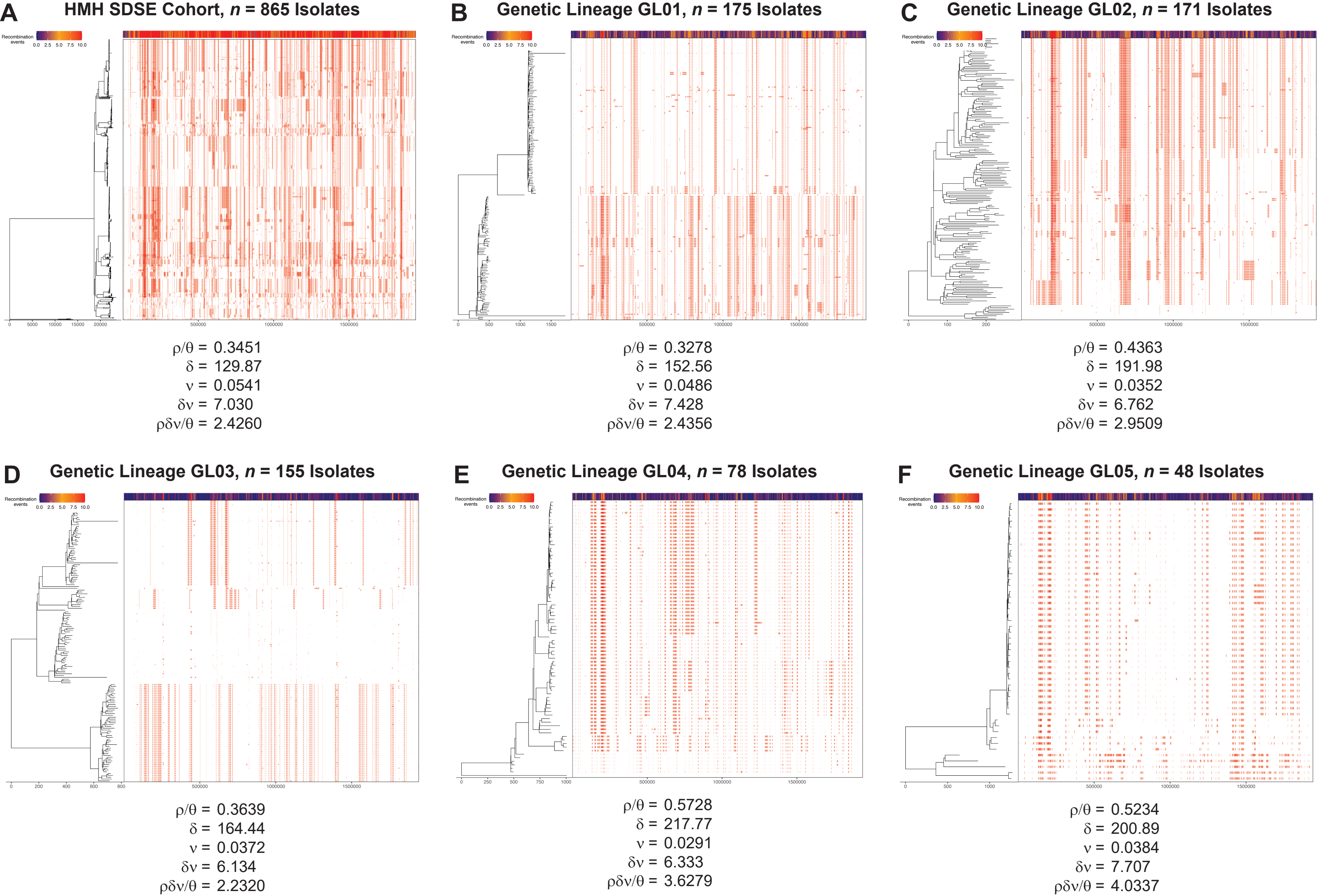

A
HMH SDSE Cohort, n = 865 Isolates
Recombination events
0.0 2.5 5.0 7.5 10.0
ρ/θ = 0.3451
δ = 129.87
ν = 0.0541
δν = 7.030
ρδν/θ = 2.4260
B
Genetic Lineage GL01, n = 175 Isolates
Recombination events
ρ/θ = 0.3278
δ = 152.56
ν = 0.0486
δν = 7.428
ρδν/θ = 2.4356
C
Genetic Lineage GL02, n = 171 Isolates
Recombination events
ρ/θ = 0.4363
δ = 191.98
ν = 0.0352
δν = 6.762
ρδν/θ = 2.9509
D
Genetic Lineage GL03, n = 155 Isolates
Recombination events
ρ/θ = 0.3639
δ = 164.44
ν = 0.0372
δν = 6.134
ρδν/θ = 2.2320
E
Genetic Lineage GL04, n = 78 Isolates
Recombination events
ρ/θ = 0.5728
δ = 217.77
ν = 0.0291
δν = 6.333
ρδν/θ = 3.6279
F
Genetic Lineage GL05, n = 48 Isolates
Recombination events
ρ/θ = 0.5234
δ = 200.89
ν = 0.0384
δν = 7.707
ρδν/θ = 4.0337
